# Does the index in Morse taper connection affect the abutment stability? An in vitro experimental study

**DOI:** 10.1371/journal.pone.0298462

**Published:** 2024-03-08

**Authors:** Valentina Paz Goyeneche, Guillermo Castro Cortellari, Fernando Rodriguez, Piedad N. De Aza, Eleani Maria da Costa, Antonio Scarano, Nilton De Bortoli Júnior, Sergio Alexandre Gehrke

**Affiliations:** 1 Departament of Implantology, Bioface/PgO/UCAM, Montevideo, Uruguay; 2 Departament of Bioingenieria, Universidad Miguel Hernández de Elche, Alicante, Spain; 3 Department of Materials Engineering, Pontificial Catholic University of Rio Grande do Sul, Porto Alegre, Brazil; 4 Department of Innovative Technologies in Medicine & Dentistry, University of Chieti-Pescara, Chieti CH, Italy; 5 Departament of Implantology, Paulista University, Paulista University (UNIP), São Paulo, Brazil; 6 Department of Biotechnology, Universidad Católica de Murcia (UCAM), Murcia, Spain; International Medical University, MALAYSIA

## Abstract

The present study compared three different implant and abutment sets of type Morse taper (MT) connection, with- and without-index, were analyzed regarding their mechanical behavior without and with cyclic load application simulating the masticatory function. Ninety implant and abutment (IA) sets were used in the present study, divided into three groups (n = 30 samples per group): Group A, Ideale solid straight abutment (one piece) without index; Group B, Ideale abutment with an angle of 30-degree (two pieces) without index; Group C, Ideale abutment with an angle of 30-degree (two pieces) with index. The abutment stability quotient (ASQ) values, detorque value and rotation angle were measured before and after the cycling load. Twenty IA sets of each group were submitted to mechanical load at 360,000 cycles. The ASQ without load were 64.7 ± 2.49 for the group A, 60.2 ± 2.64 for the group B, 54.4 ± 3.27 for the group C; With load were 66.1 ± 5.20 for the group A, 58.5 ± 6.14 for the group B, 58.9 ± 2.99 for the group C. Detorque values were lower in groups B and C compared to group A (p < 0.05). In conclusion, the presence of the index did not influence the stability values. However, solid straight abutments (group A) showed higher values of stability compared to groups of angled abutments (groups B and C).

## Introduction

Different interfaces between implant and abutment (IA) were developed with the aim of achieving better performance and safety during masticatory loads, providing adequate clinical longevity [1, 2]. Morse taper (MT) connections provide better adaptation between the IA sets, decrease and/eliminating the spaces between them (gaps), reducing peri-implant bone resorption levels, and minimizing micromovements between IA sets [3, 4]. Also, loosening of sets is less frequent in MT connections compared to other connection types [5, 6]. Conical connections have an internal design that, after installation of the abutment, promote the union between the surfaces (internal of the implant and external of the abutment), helping in the mechanical properties and stability of the prosthetic abutment. Thus, increasing resistance to separation forces by the coefficient of friction between pieces (implant and abutment) [7]. After the preload during the installation of the abutments, which is done by applying a torque determined by the manufacturer, these sets should remain stable during the application of masticatory loads. However, as described in previous studies [8], in most systems (IA sets) there is accommodation of the parts during the application of loads that can change the initial torque applied and, consequently, change the stability of the sets. In addition, non-tapered IA sets (external and internal hexagon connections) may exhibit micromovements during the application of forces [9].

Originally, the MT implant systems did not have any type of anti-rotational portion for positioning the abutment, even in the case of angulated abutment, which determines that it is positioned/torqued and is no longer removed. However, more recently, a fitting called a prosthetic index was inserted in this connection, being especially used in the case of single implants in which the insertion of the prosthesis must be guided to a specific position that provides a perfect fit. These indexed IA sets are secured by a through screw, facilitating correct positioning of the abutment during insertion [10]. However, it is not yet clear whether the presence of the index can alter the mechanical resistance of the MT implant systems, due to the reduction in the extension of the implant/abutment contact, or if it alters the friction between the pieces. Furthermore, Zhang et al. [10] showed that indexed abutments create zones with stress accumulation that can generate biomechanical complications.

On the other hand, other studies evaluated whether the inclusion of the index could affect bacterial sealing and the resistance of the IA sets, since it results in a decrease in the contact area in the conical portion of the implant/abutment. Based on methodologies for evaluating bacterial microleakage and resistance to fracture, the authors observed that the presence of the index did not compromise the performance of MT implants [8, 9, 11].

Resonance frequency analysis (RFA) was initially developed as a non-invasive method to measure the stability of implants during their osseointegration [12]. However, with the availability of sensors for multi-unit abutments and the development of multifunctional abutments, such as the Ideale abutment, the possibility of also measuring the stability of these abutments using this method has emerged (RFA) [13]. On the other hand, the use of in vitro tests is important because different samples (abutment designs) can be analyzed under the same conditions, unlike clinical studies (patients) where in each situation there are several variables involved [14]. Therefore, this type of test can bring relevant information to clinical practice, providing professionals with confidence in its indication for rehabilitative treatments.

In the present in vitro study, three different IA sets of type MT connection, with- and without-index, were analyzed regarding their mechanical behavior with and without the application of cyclic loads simulating masticatory loads. For this, measurements were taken of the abutment detorque (solid) and of the fixation screws (two-piece abutments), abutment stability and angle of rotation of each type of abutment to reach the recommended torque. The main hypothesis was that the presence of the index on the abutment could negatively influence the achievement of stability between the IA sets. The second hypothesis was that the torque loss of the fixing screw in two-piece abutments does not influence the maintenance of the IA sets union.

## Materials and methods

### Materials used

**Fig 1:**
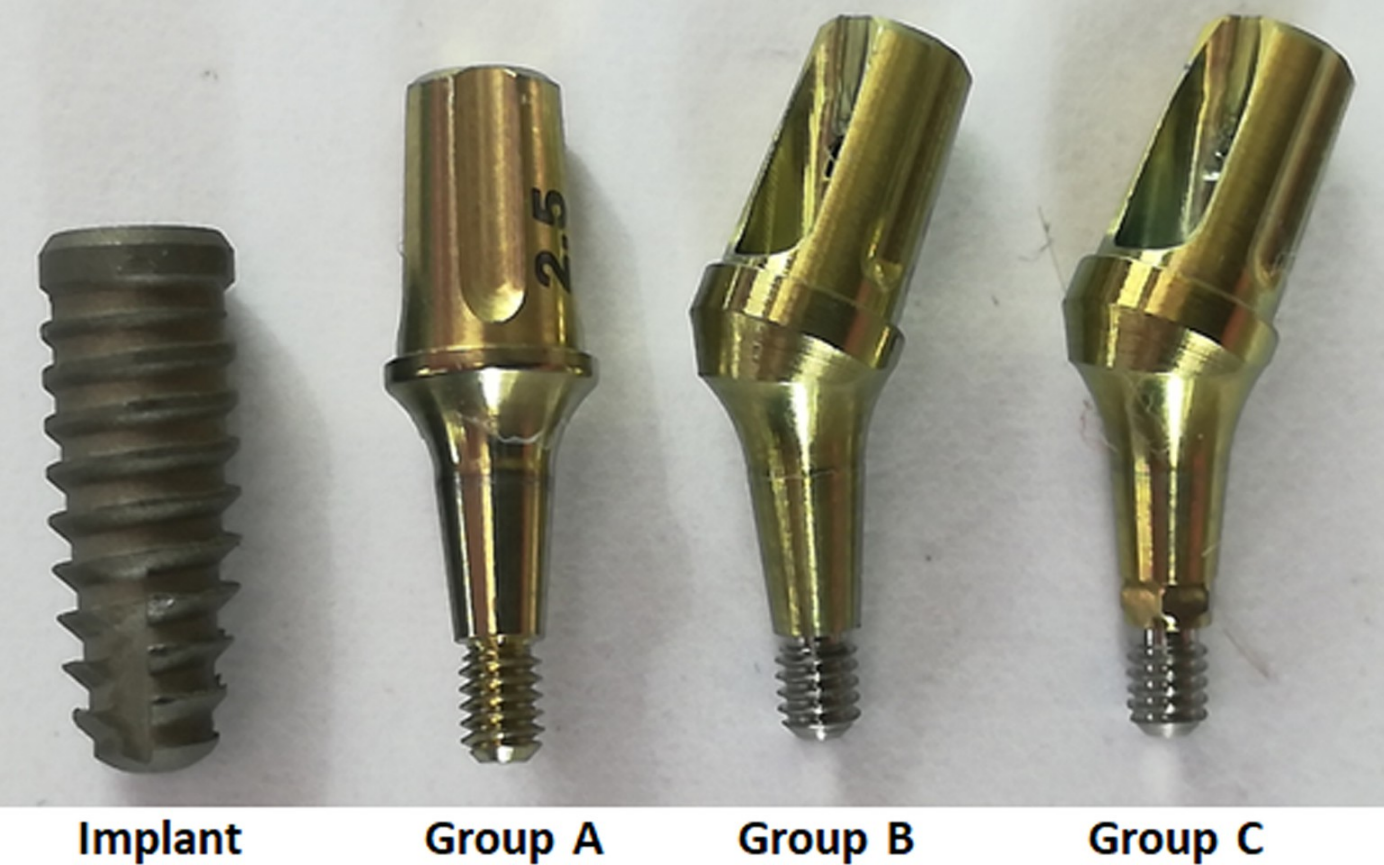
Representative image of the implant and abutments used.

A total of ninety IA sets were used in the present study, which were divided into three groups according to the design of each abutment used (n = 30 samples per group): Group A, where Ideale solid straight abutment (one piece) without index were used; Group B, where Ideale abutment with an angle of 30-degree with two pieces (fixation screw and abutment) without index were used; Group C, where Ideale abutment with an angle of 30-degree with two pieces (fixation screw and abutment) with index were used. All connection tested presented an angulation of the IA sets at 11.5-degree. Ninety DuoCone implants used were tapered and had dimensions of 4 mm in diameter and 11 mm in length. **Fig 1** shows a representative image of the abutments and the implant used. All materials (abutments and implants) used are manufactured and marketed by the company Implacil De Bortoli (São Paulo, Brazil).

**Fig 2:**
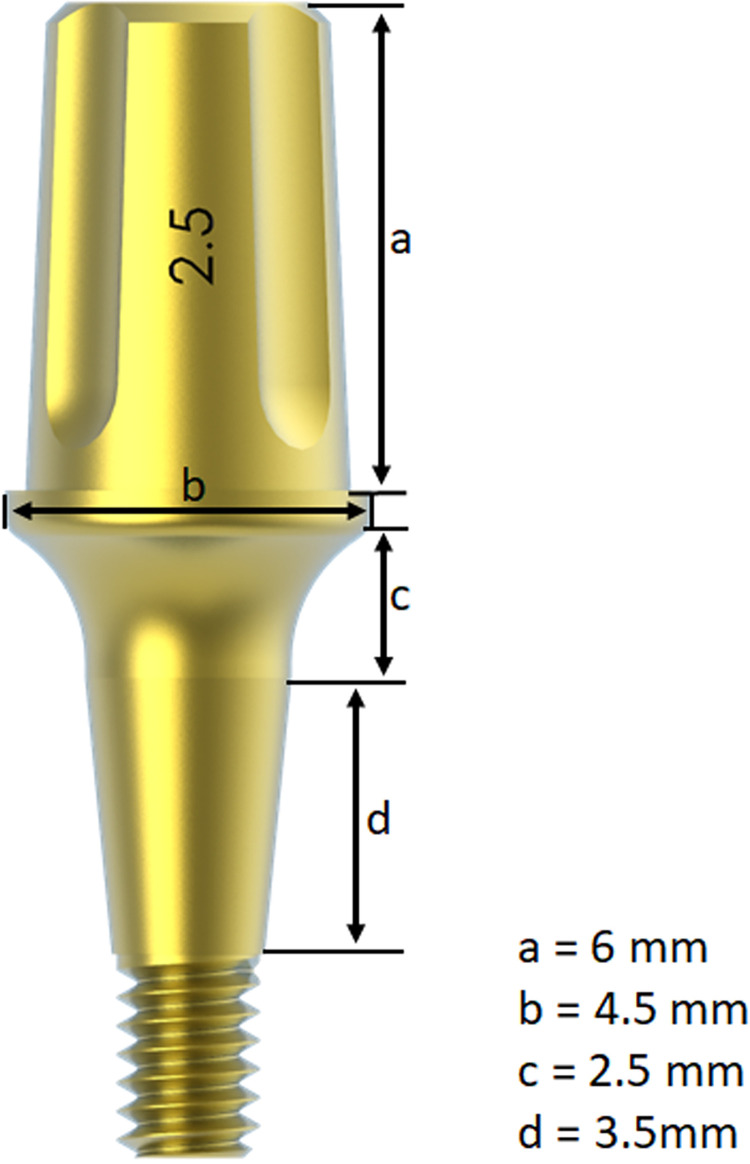
Image demonstrating the dimensions in the main parts of the abutment.

All abutments of three groups were manufactured in titanium F67 grade IV and were anodized after the machining. The mean value of roughness (Sa) provided by the manufacturer for all abutments was 2.21 ± 0.27 μm. All Ideale abutments were produced for Morse taper connection implants and present the same dimensions, as shown in **Fig 2**.

For the sample size calculation, the software SigmaStat 4.0 (Systat Software Inc, San Jose, USA) was used, and a power level of 85% was considered to obtain a P value of 0.05. For a desired power level of 85% with differences between each group’s means and standard deviations, the minimum sample size for each group under each condition was 8 sets (implant and abutment). Thus, we used 10 sets for each group and condition.

### Samples preparation

**Fig 3:**
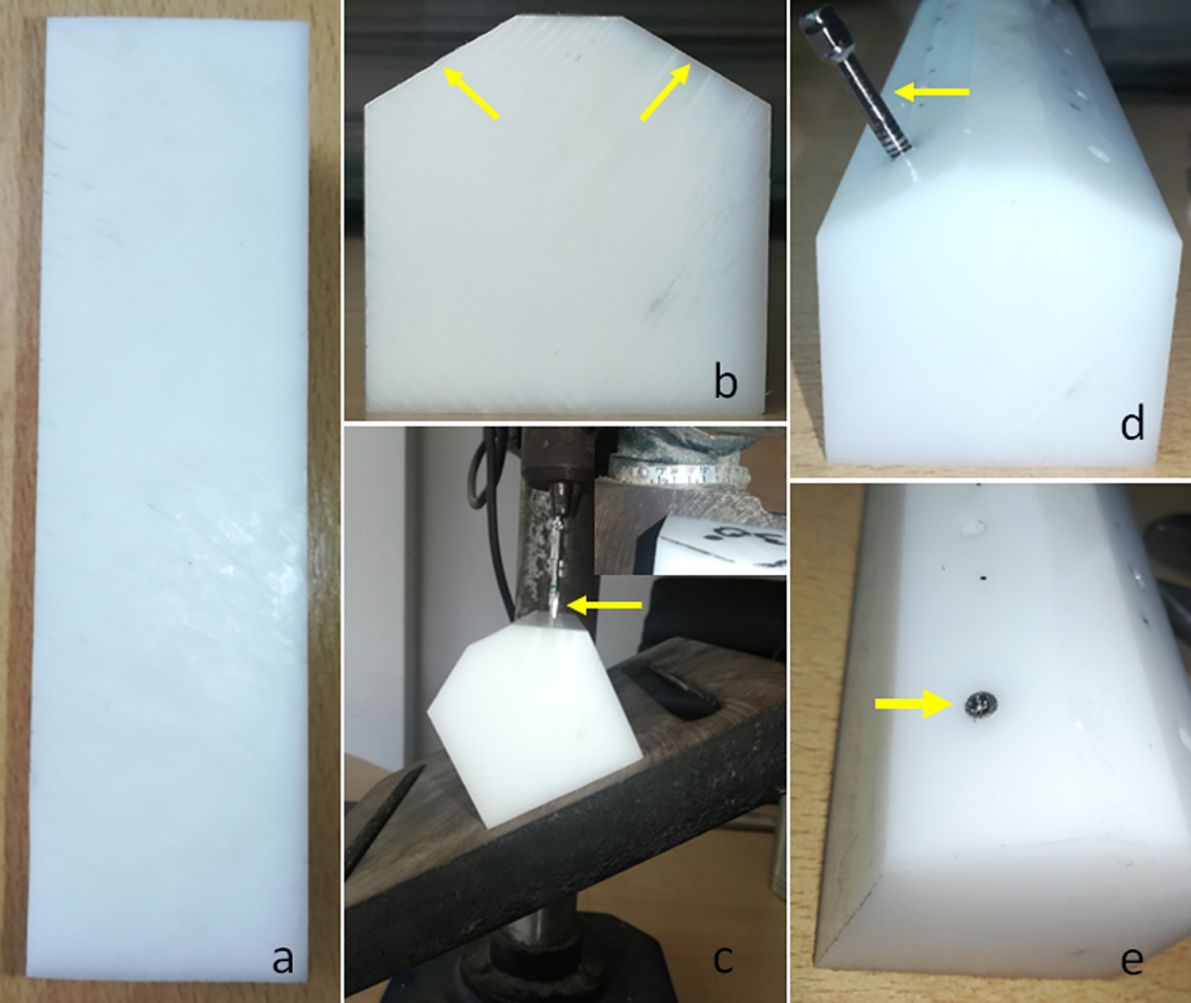
(a) Image of the block made to support the IA sets during the test; (b) the arrows indicating the cutting of the two edges in each block with an angle of 30 ± 2 degrees; (c) representative image of the drilling moment with the base-table angled at 30 degrees to obtain the desired inclination of the implant; (d) arrow indicating the implant installation driver and its inclination; (e) arrow showing the implant positioned inside the block at the level of its platform.

Initially, nine rectangular blocks were made in epoxy resin (G4, Polipox, São Paulo, Brazil) with dimensions of 40 x 40 x 150 mm (**Fig 3A**). These blocks were taken to an angle saw and an angled cut of 30 ± 2 degrees was made on two edges of each block (**Fig 3B**). Then, the perforations were made using the drill sequence indicated by the manufacturer on drilling equipment with the base-table angled at 30 degrees, obtaining the desired inclination for the implants (**Fig 3C**). Finally, all DuoCone implants were manually inserted into the blocks up to the platform level (**Fig 3D and 3E**) and, the abutments were screwed and torqued. The angled positioning of the implants followed the recommendation of the ISO 14801:2015 standard [15], which considers this angle to be the most clinically critical.

**Fig 4:**
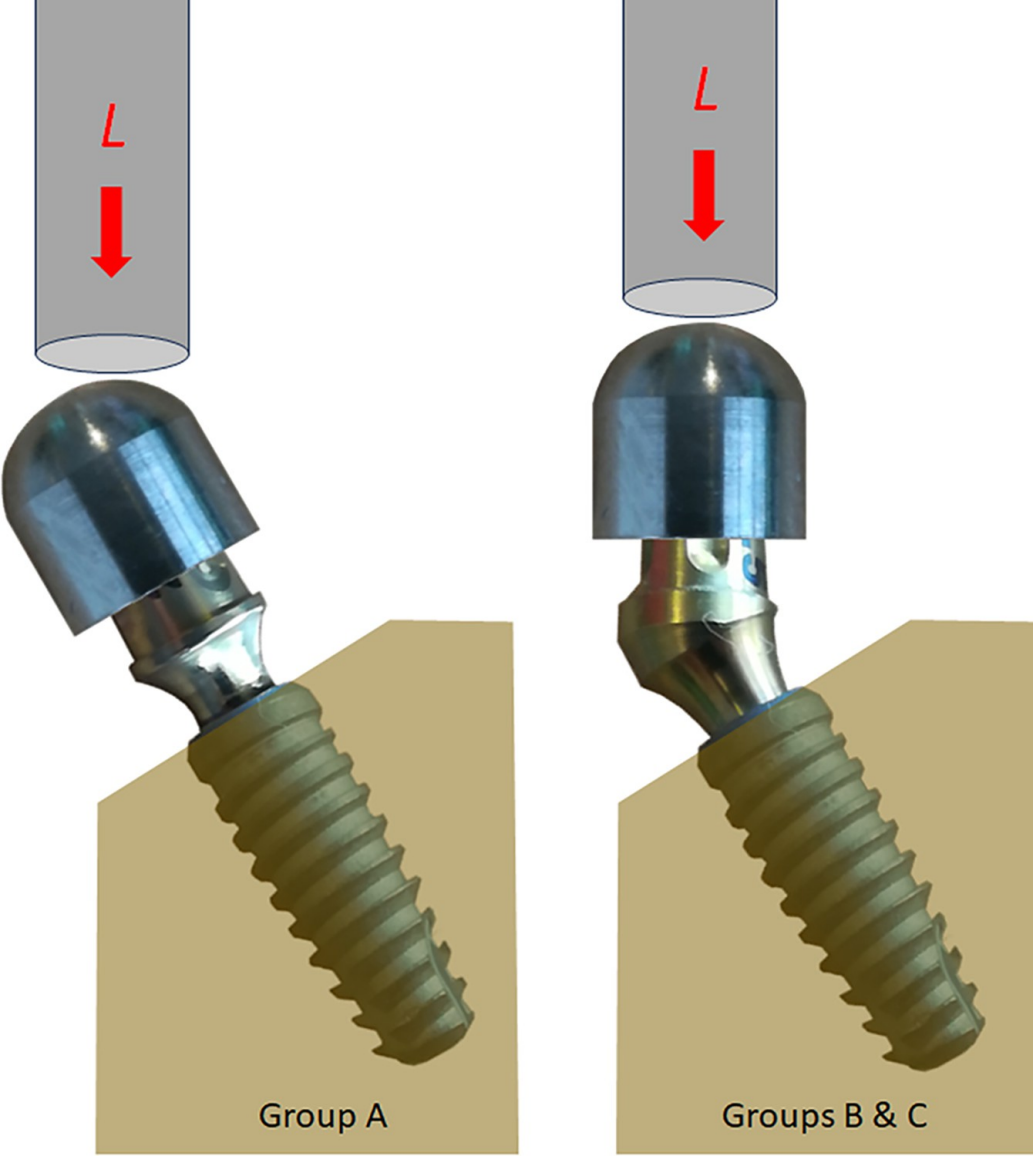
Schematic image to show the positioning of the implants in the blocks and the direction of the loads applied to the samples of the three groups.

A metallic hemisphere was prefabricated for each abutment and positioned on them to receive the loads applied during the test, to avoid damaging the abutment. These details follow the standard ISO 14801:2015 [15]. **Fig 4** shows the positioning of the implants in the blocks and the direction of the loads applied to the samples of the three groups.

The abutments were installed in each implant and were torqued in accordance with the manufacturer’s recommendations: group A = 30 Ncm, group B and C = 20 Ncm. Torque was applied using computerized torquemeter equipment model CME-30Nm (Técnica Industrial Oswaldo Filizola Ltda, São Paulo, Brazil) with a speed of 3 mm/min, with an angular measurement with resolution of 0.002-degree. Ten minutes after the first torque, all samples were retorqued [16]. The angle of rotation during the application of the second torque (retorque) and the retorque in 10 samples of each group after the mechanical load application was recorded for analysis and comparison between groups.

### Mechanical load cycles

Twenty samples of each group were submitted to the mechanical load cycles with an application of 360,000 cycles with a controlled non-axial force of 150 N at 4 Hz of frequency using a mechanical machine (Biocycle V2, BioPDI, São Carlos, Brazil), following previous studies published [4, 17]. The samples stayed immersed in water with a controlled temperature at 37 ± 2°C during the load cycles application.

### Stability measurement

**Fig 5:**
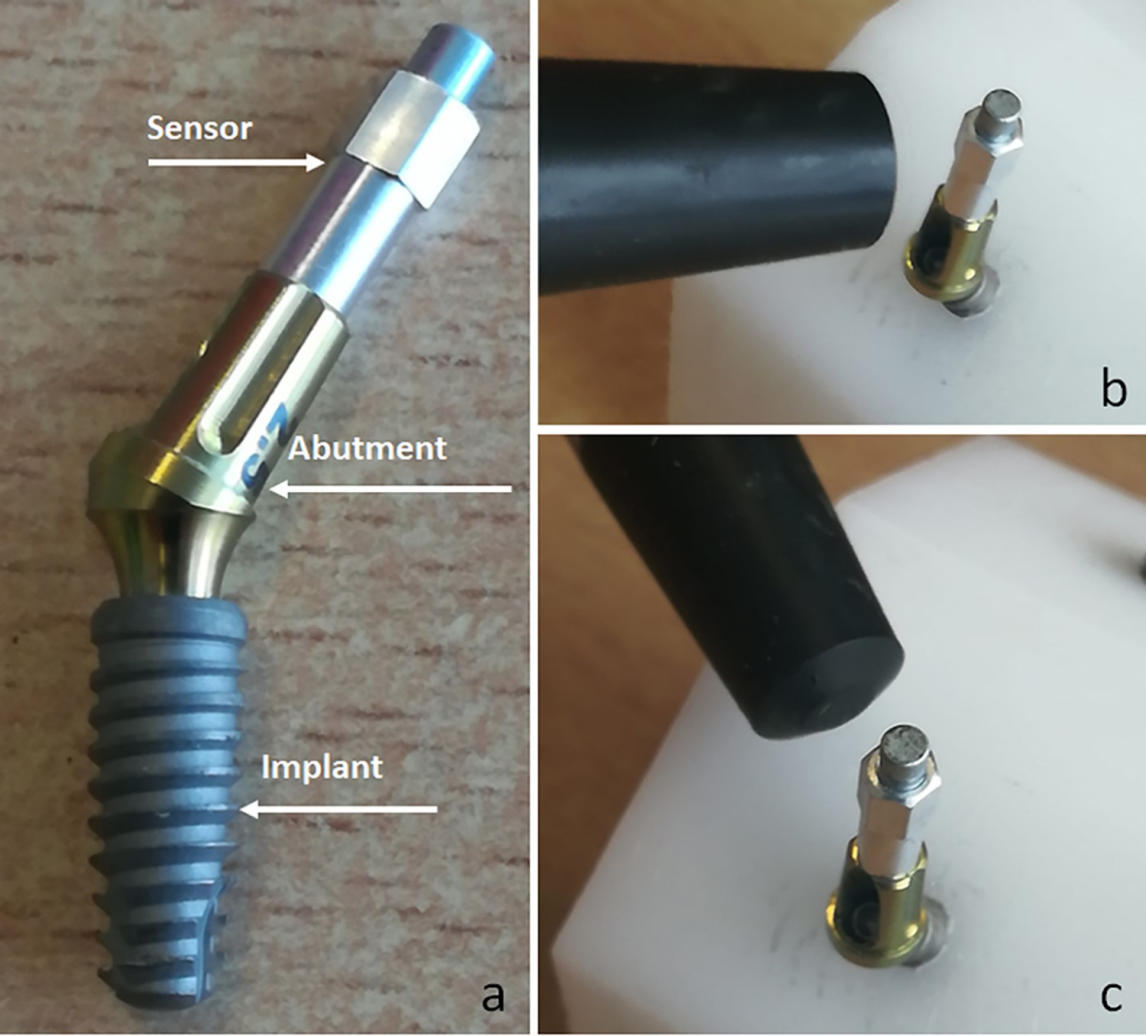
Representative image of the IA set, and the magnetic sensor installed (a). ASQ measurement in the frontal direction (b) and in lateral direction (c).

One of the main features of the Ideale abutment is the ability to receive both screw-retained and cemented crowns, as it has an internal thread on the upper part like a multi-unit abutment, which makes it possible to use this sensor to measure stability [13, 18]. Then, after completion of cyclic loading, all loaded and unloaded specimens were evaluated for abutment stability using the Osstell Mentor device (Integration Diagnostics AB, Göteborg, Sweden). For this, the magnetic sensor type 25 (Smartpeg, Integration Diagnostics AB, Göteborg, Sweden) was screwed onto each abutment and the abutment stability quotient (ASQ) was measured in two different directions (**Fig 5**).

### Detorque measurements

After stability measurements, all samples of each group were subjected to abutment detorque in group A and abutment fixation screws in groups B and C, using the computerized torquemeter equipment model CME-30Nm (Técnica Industrial Oswaldo Filizola Ltda, São Paulo, Brazil), the same equipment used for applying torque. The maximum value obtained was recorded and used for comparisons between groups. Due to the difference in applied torque values, as recommended by the manufacturer, for each group the difference between the applied value and the maximum value obtained in detorque was calculated, and these values were used for evaluations and comparisons between groups.

### Statistical analysis

Data collected in each test were statistically compared using the Bonferroni’s multiple comparison test to detect possible differences between groups, always considering p < 0.05 as statistically significant. All analyzes were performed using GraphPad Prism 5.01 software (GraphPad Software Inc., San Diego, USA). Possible correlations between abutment stability and abutment detorque values were tested using the Pearson correlation test.

## Results

### Abutment stability results

**Fig 6:**
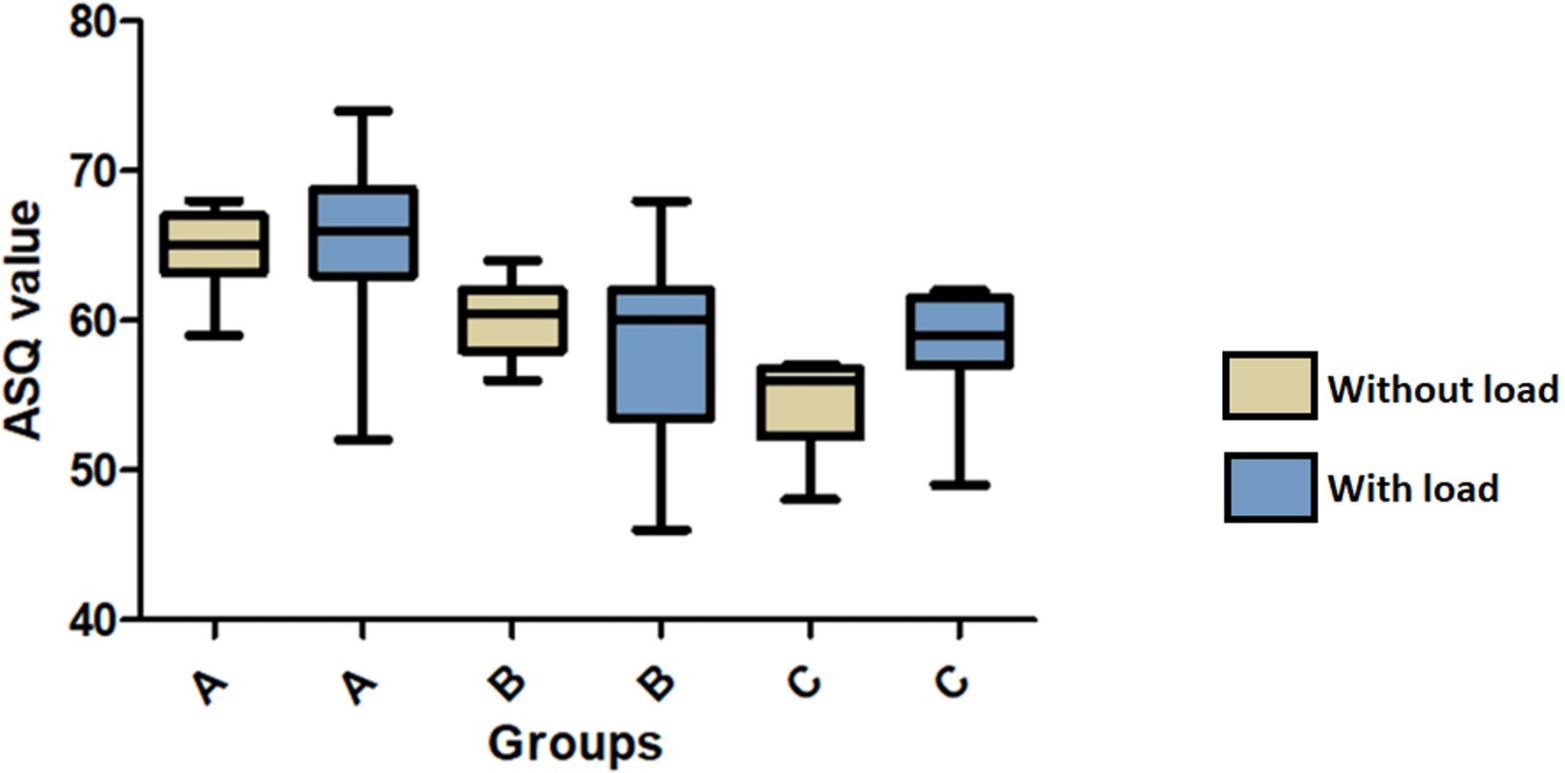
Box plot graphs shows the data values obtained for the abutment stability quotient (ASQ) at the three groups without- and with-load.

With the purpose of increasing the security of the stability values measured on the abutments, the stability of all implants inserted in resin blocks were measured before the abutments installation and resulted in a high value in all samples (ISQ = 85 ± 0.9). The mean and standard deviation of ASQ values obtained for the groups without load application were: 64.7 ± 2.49 for the group A, 60.2 ± 2.64 for the group B, 54.4 ± 3.27 for the group C. While, in the samples that received loads, the values obtained were as follows: 66.1 ± 5.20 for the group A, 58.5 ± 6.14 for the group B, 58.9 ± 2.99 for the group C. These data distribution is presented in the graph of **Fig 6**. The statistical comparison intragroup only shows difference for the group C (p < 0.0001). **Table 1** shows the statistical comparison intergroups using the Bonferroni’s multiple comparison test.

**Table 1:** Statistical comparison intergroups using the Bonferroni’s multiple comparison test.

	Without load			With load		
Groups comparison	Mean Diff.	*P*-value	95% CI of diff	Mean Diff.	*P*-value	95% CI of diff
A vs B	4.550	< 0.0001*	2.350 to 6.750	7.650	0.0002*	3.783 to 11.52
A vs C	10.35	< 0.0001*	8.150 to 12.55	7.200	< 0.0001*	3.333 to 11.07
B vs C	5.800	< 0.0001*	3.600 to 8.000	-0.450	0.9240	-4.317 to 3.417

Diff. = difference; CI = confidence interval

* = statistically different.

### Detorque results

**Fig 7:**
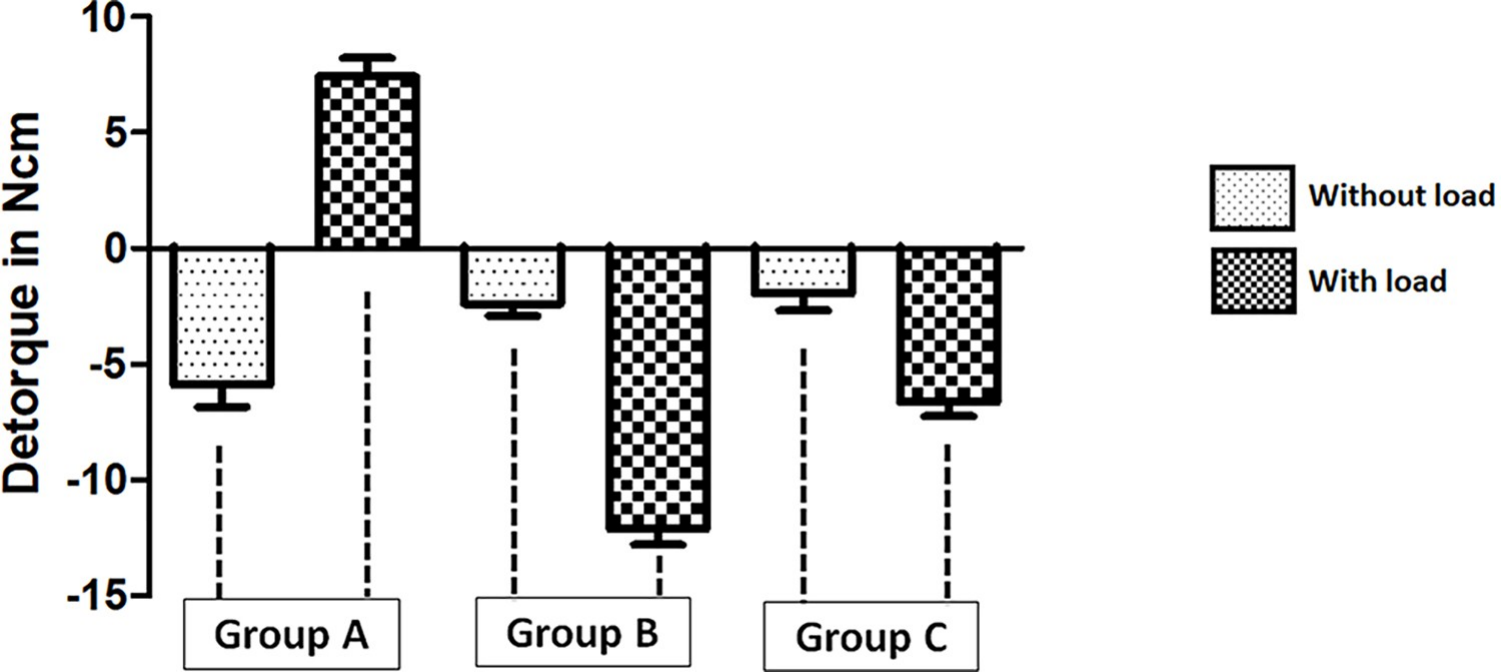
Bar graphs showing the distribution of data obtained after calculating the difference between the applied torque and the obtained torque value, with and without load application.

The detorque values obtained of all group samples without the load application, presented values below the initial torque value, and group A presented a mayor loss of torque (- 19.7%) compared to group B (- 12%) and group C (- 9.5%). However, after applying the loads, the group A presented a detorque value superior at the initial torque (+ 24.7%), while group B (- 33%) and group C (- 64%) had values far below the initial torque. **Fig 7** graphically presents the distribution of the data obtained after calculating the difference between the applied torque and the obtained detorque value, without and with load application.

In the intragroup statistical analysis, all groups showed statistical differences (p < 0.0001). The statistical analysis between groups is presented in **Table 2**.

**Table 2:** Statistical comparison intergroups using the Bonferroni’s multiple comparison test for the detorque values.

	Without load			With load		
Groups comparison	Mean Diff.	*P*-value	95% CI of diff	Mean Diff.	*P*-value	95% CI of diff
A vs B	-3.500	0.0113*	-6.239 to -0.761	19.53	< 0.0001*	17.01 to 22.05
A vs C	-3.950	0.0091*	-6.689 to -1.211	14.05	< 0.0001*	11.53 to 16.57
B vs C	-0.450	0.7045	-3.189 to 2.289	-5.480	< 0.0001*	-8.002 to -2.958

Diff. = difference; CI = confidence interval

* = statistically different.

### Angle results

**Fig 8:**
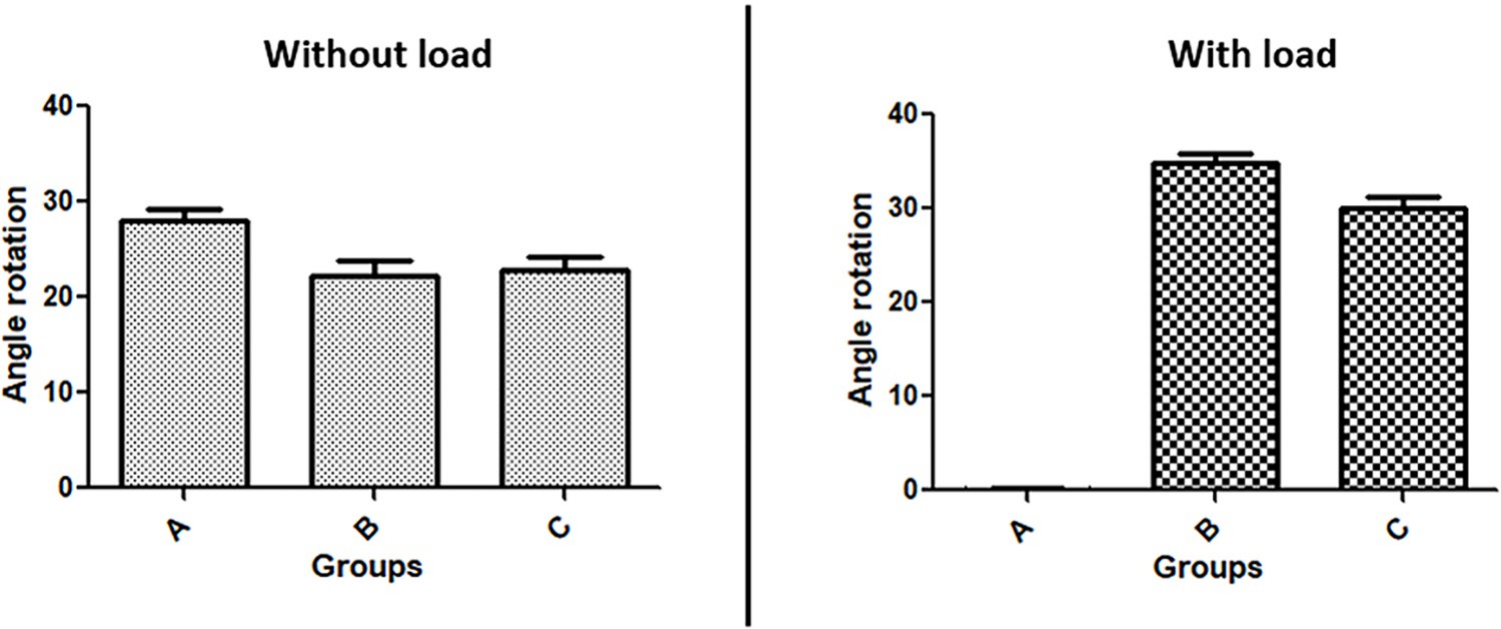
Bar graphs presenting the data distribution of the angle rotation obtained in each group, without and with load application.

The angle rotation values obtained in the retorque (10 minutes after the first torque) of all group samples, without load application, shows a superior value to the group A (24.1 ± 3.04 degrees) in comparison to the group B (24.1 ± 3.04 degrees) and group C (18.1 ± 2.31 degrees). However, after applying the loads, the samples of group A did not show rotation, while group B shows 34.9 ± 3.11 degrees and group C 30 ± 3.84 degrees. **Fig 8** graphically presents the distribution of the angle rotation obtained in each group, without and with load application. **Table 3** shows the statistical comparison intergroups using the Bonferroni’s multiple comparison test.

**Table 3:** Statistical comparison intergroups using the Bonferroni’s multiple comparison test for the angle rotation values.

	Without load			With load		
Groups comparison	Mean Diff.	*P*-value	95% CI of diff	Mean Diff.	*P*-value	95% CI of diff
A vs B	5.720	0.0203*	0.6887 to 10.75	-34.76	0.0002*	-38.02 to -31.50
A vs C	5.060	0.0230*	0.02871 to 10.09	-29.93	0.0002*	-33.19 to -26.67
B vs C	-0.6600	0.5957	-5.691 to 4.371	4.830	0.0057*	1.573 to 8.087

Diff. = difference; CI = confidence interval

* = statistically different.

### Correlation analysis

No correlation was detected between the ASQ values and abutment detorque values, ASQ values and rotation angle values, in both conditions tested. However, between in detorque values and rotation angle values a strong correlation was detected (*r* = 0.8453).

## Discussion

Constant design modifications have been proposed to improve the long-term biomechanical behavior of implant systems used for the rehabilitation of missing teeth. As they are parts of relatively small dimensions, but that receive relatively large and constant efforts (loads), they need high precision and stability between their components to adequately support this condition. The results obtained in the present study showed a similar stability pattern (ASQ) for both groups of abutments with and without index (B and C groups), in the two conditions tested (with and without application of loads). However, the abutments in group A showed stability values (ASQ) superior to those in groups B and C in both tested conditions (with and without application of loads). Thus, based on the results, the initial hypothesis that the presence of the index negatively influences stability between IA sets might be rejected. The data suggests that the index may not have the expected detrimental effect. Moreover, the second hypothesis that the torque loss of the fixing screw in two-piece abutments does not influence the maintenance of the IA sets union was confirmed.

The presence of micromovements between the parts (implant and abutment) can generate changes in the contact surfaces (interfaces), as well as in the abutment fixation screw [19]. This factor can cause loss of preload, resulting in reduction of contact forces between the abutment cylinder and the implant body and, consequently, loosening of the fixation screw [20]. In addition, the existing micromovements in the AI sets allow the passage of bacteria and their generated fluids, which can cause inflammation of different intensities in the peri-implant tissues [21]. Due to the importance of this, our study sought to evaluate the stability of abutments with Morse taper connection with different designs, as there were no similar publications in the literature so far. The Ideale multifunctional abutment used in our study, which allows the creation of cemented and/or screw-retained crowns [13], made it possible to obtain stability measurements without removing the abutment from its position.

Obtaining abutment micromovement values was only possible in tests performed in vitro using 3D microtomography images or radiographic images [5, 22, 23]. In addition, other in vitro techniques were used to analyze the interface between the implant and the abutment, most of them using scanning electron microscopy and destroying the samples through the cuts made for these evaluations [15, 16, 24]. Since the creation of non-invasive methods for measuring the stability of implants through resonance frequency analysis (RFA), new possibilities for determining the degree of osseointegration have been introduced into clinical practice [25, 26]. More recently, with the elaboration of a sensor that adapts to the thread system of transmucosal abutments, and with the appearance of the Ideale multifunctional abutments, which allow the screwing of this sensor, it became possible to measure the stability through this method (RFA) of the abutments. Other authors used this method to assess, for example, whether the height of the abutment could influence the stability values, and the results showed that the values may vary with the increase in the abutment height [27]. However, other studies have shown that varying the height of the abutments does not change the values measured by Osstell [28, 29]. In our study, abutments of similar heights were used, with a difference of 1 mm between the abutments in group A and the abutments in groups B and C. However, when straight abutments were compared with angled abutments, angled abutments had lower stability values measured by RFA [27], corroborating our results. On the other hand, when we compare the results obtained in this study with the results recently published by our research group, we observe a proximity in the measured stability values after the application of cyclic loads [18].

The friction in the conical connections is responsible for the stability of the abutments. In this way, other authors sought to assess whether the presence of an index on the implant could change the friction values between the abutment and the implant, as the index reduces the extent of contact between the parts, and the results of this study showed that there was no reduction in friction on the tested IA sets [30]. In our study, all implants used had an index, and the intention was to assess whether the presence of an index on the abutment could have a negative effect on the stability of the abutments. Our results showed that there was no difference in measured stability values between indexed and non-indexed abutments (groups B and C). Interestingly, the study found that the abutments in group A exhibited higher stability values (ASQ) compared to groups B and C. This difference in stability was consistent across both tested conditions (with and without load application). Group A seems to have shown the best performance in terms of stability among the three groups.

Regarding the abutment detorque in group A, similar results were obtained in previous studies before and after application of loads [15]. However, in this previous study, it is important to point out that implants without an index were used, which corroborates the findings cited by Michelon and collaborators [30], that the presence of an index in the implants does not alter the friction values of this type of solid straight abutment. While, in the evaluation of the detorque of the fixation screws of groups B and C, both showed values below the initial torque applied: without the application of loads, the value of torque loss was small, unlike the values obtained after the application of loads, which shows a bigger torque loss. However, as there was an increase in the measured values of stability (ASQ), we can conclude that the fixation screw, in this type of conical connection, does not participate as a union element between the IA sets, corroborating previous findings published by other authors [31].

The retorque angle measured in all unloaded samples of all groups showed the need for retorque after the first torque, corroborating the recommendations of Breeding et al. [32]. In the samples that received cyclic loads, we observed that in group A the angle of rotation was practically zero in all samples, coinciding with the fact that the torque of the abutments was above the initial value applied (> 30 Ncm). However, in the samples from groups B and C, the rotation angle was much higher, also coinciding with the fact that the abutment fixation screws lost a significant amount of their initial torque value (< 20 Ncm). The screw rotation angle showed a strong correlation with the obtained detorque values.

Finally, it’s important to consider any limitations of the study that might affect the generalizability of the results. For instance, the sample size, the specific conditions of the test, or other factors could impact the robustness of the findings. The study doesn’t delve into the reasons behind the varying stability among the groups. Factors like design, material, or other characteristics of the abutments might contribute to these differences. Future research could explore these aspects to better understand what’s driving the observed variations in stability.

## Conclusions

Within the limitations of this in vitro evaluation, it might be concluded that the presence of a positioning index does not influence the biomechanical stability. However, solid straight abutments (group A) showed higher values of stability compared to groups of angled two-piece abutments (groups B and C). Furthermore, based on the detorque and rotation angle values of the fixation screw in the two-piece abutments, we can conclude that the abutment stability is independent of the fixation screw in this conical connection tested.
